# Watching Parkinson’s disease with wrist-based sensors

**DOI:** 10.1038/s41746-022-00619-4

**Published:** 2022-06-13

**Authors:** James A. Diao, Marium M. Raza, Kaushik P. Venkatesh, Joseph C. Kvedar

**Affiliations:** Harvard Medical School, Boston, MA USA

**Keywords:** Biomarkers, Parkinson's disease

## Abstract

Parkinson’s disease (PD) lacks sensitive, objective, and reliable measures for disease progression and response. This presents a challenge for clinical trials given the multifaceted and fluctuating nature of PD symptoms. Innovations in digital health and wearable sensors promise to more precisely measure aspects of patient function and well-being. Beyond research trials, digital biomarkers and clinical outcome assessments may someday support clinician-initiated or closed-loop treatment adjustments. A recent study from Verily Life Sciences presents results for a smartwatch-based motor exam intended to accelerate the development and evaluation of therapies for PD.

## Main

After transforming information exchange and the global economy, consumer technology has come for healthcare. Smartwatches now have nearly half a billion active users^1^, enabling longitudinal and multimodal health monitoring at unprecedented scale. In particular, digital biomarkers and clinical outcome assessments (COAs) may improve on qualitative evaluations for conditions like Parkinson’s disease (PD). A team from Verily (formerly Google Life Sciences) developed and validated a smartwatch-based motor exam for PD that is concordant with standard disease severity ratings, responsive to medication effects, and reliable across repeat measurements^2^. A virtual motor exam could complement in-clinic visits and passive monitoring strategies to unlock more precise insights for PD treatment. Remaining challenges include integration of data streams across complementary monitoring techniques and alignment of digital measurements with outcomes most meaningful to patients.

Co-sponsored by Verily, the Personalized Parkinson’s Project (PPP) is a prospective single-center study of patients with early-stage PD in the Netherlands^3^. Participants are monitored at 4-month intervals via on-site clinical assessments and continuously via the Verily Study Watch. Using accelerometer data from the Study Watch, the authors developed a PD virtual motor exam (PD-VME) consisting of eight self-guided tasks. Each task corresponds to elements of the Movement Disorder Society-Unified Parkinson’s Disease Rating Scale (MDS-UPDRS) Part III exam^4^, currently the “gold-standard” for motor evaluation of PD.

Digital instructions guided participants through the motor exam: once under clinical observation and then twice weekly at home. In total, 370 participants logged 22,668 virtual exams over 70 weeks. Exam measures, such as lateral tremor acceleration for assessing resting tremor, exhibited moderate-to-strong correlation with consensus disease severity ratings (MDS-UPDRS III), test-retest reliability consistent with that of MDS-UPDRS III^5^, and small-to-medium effect sizes for fast-acting PD medications. Other investigated measures included arm-twisting amplitude for measuring upper extremity bradykinesia and arm swing acceleration for measuring gait impairment.

Relative to in-clinic exams, virtual exams offer more frequent and convenient monitoring in natural living environments. Notably, PD-VMEs conducted in the clinic were not always concordant with PD-VMEs conducted at home using the same protocol. This suggests that clinical observation may not faithfully capture the real-world experiences of PD patients, but could also indicate increased protocol deviations at home. Regardless, in-clinic exams may complement virtual exams by providing access to medical staff to confirm disease diagnoses, identify major protocol deviations, and order further testing.

Virtual exams may also synergize well with passive monitoring. On the one hand, virtual exams are better able to assess intent to move when evaluating symptoms like bradykinesia. On the other hand, passive monitoring enables higher temporal resolution^6^ and may impose fewer burdens on patients. It does require consistent use, which appears achievable given the average daily wear time of 22.1 hours in the PPP study. Virtual exams only require interaction twice a week. Still, the completion rate of PD-VMEs was imperfect, declining from 80% in week 1 to 40% in week 52. These trade-offs indicate that virtual exams may offer the greatest promise when implemented alongside in-clinic visits and passive monitoring strategies.

A robust evidence base for clinical utility is needed for regulatory approval and commercial uptake. Objectives for future work were outlined in a recent exchange between Verily^7^ and the US Food and Drug Administration (FDA)^8^, in which the agency called for measures that are more “representative of daily life functioning” and more “meaningful to patients,” such as changes to speech, eating, or dressing. Integration of questionnaire components and non-motor tasks may satisfy this request. Other improvements may come from combining motor exam measures. For example, the interaction of arm-twisting rate multiplied by arm-twisting amplitude was more predictive than either variable alone. Machine learning methods supervised by clinical disease scores or medication state may identify and validate more complex associations. Future research may also integrate measures from other sensors, such as microphones for speech^9^, video recordings for facial expression^10^, and insoles for postural instability^11^.

The field is moving quickly^12^. To expand beyond early-stage PD in the Netherlands, Verily is studying moderate-to-severe PD in Japan and has plans for study deployment across Europe and North America^7^. Public-private partnerships continue to contribute data toward public use^13^, as Verily has promised for pseudonymised PPP study data^3^. Pharma, payers, and other industry stakeholders continue to seek consensus on best practices^14^ while proposed digital endpoints continue to proliferate^15^. For the elusive task of reliable PD assessment, the virtual motor exam contributes a valuable middle ground between isolated clinical exams and constant passive monitoring. As Verily’s team prepares future submissions to the FDA, the PD-VME will continue to face scrutiny of its validity and ultimate benefit to patients. The bar is rightfully high. But with proper research and validation, remote sensors may soon provide a more powerful and precise set of tools for studying this devastating disease.

## Data Availability

No computer code or datasets were produced or analyzed for this article.
